# The Effect of a Secondary Stressor on the Morphology and Membrane Structure of an Already Challenged Maternal and Foetal Red Blood Cell Population

**DOI:** 10.3390/ijms26010333

**Published:** 2025-01-02

**Authors:** Ágnes Ferencz, Payal Chakraborty, Csaba Papp, András Teleki, Krisztina Dugmonits, Hajnalka Orvos, Attila Gácser, Edit Hermesz

**Affiliations:** 1 Department of Biochemistry and Molecular Biology, Faculty of Science and Informatics, University of Szeged, H-6701 Szeged, Hungary; 2 HCEMM-USZ Fungal Pathogens Research Group, Department of Biotechnology and Microbiology, Faculty of Science and Informatics, University of Szeged, H-6701 Szeged, Hungary; 3 Department of Obstetrics and Gynaecology, Albert Szent-Gyorgyi Medical School, University of Szeged, H-6701 Szeged, Hungary

**Keywords:** *Candida* species, cell death, hemolysis, maternal and foetal/neonatal red blood cells, membrane damage, smoking

## Abstract

The red blood cell (RBC) membrane is unique and crucial for maintaining structural–functional relationships. Maternal smoking induces significant changes in the morphological, rheological, and functional parameters of both maternal and foetal RBCs, mainly due to the continuous generation of the free radicals. The major aim of this study was to follow the consequences of a secondary stressor, like fungal infection, on the already compromised RBC populations. The impact of *Candida* infection, a growing health concern, was investigated on four blood sample groups: mothers and their neonates originating from non-smoking versus smoking populations. Here, we searched for phenotypical and molecular markers that precisely reflected the effect of *Candida* infection on the RBC membrane; this included the level of hemolysis, appearance of morphological variants, formation of the lipid peroxidation marker 4-hydroxyl-nonenal, arrangement of the Band 3 molecules and activation of the Caspase 3. In most of the examined cases, the fungal infection increased the adverse symptoms induced by smoking, indicating a general stress response, likely due to an altered redox state of the cells. However, we were able to identify an atypical phenotype (clustered populations with shrinkage and membrane blebbing) in both the non-smoking and smoking populations, which might be a unique marker for *Candida* spp. infection.

## 1. Introduction

Epidemiological and toxicological studies undoubtedly suggest that intensive tobacco smoking causes many health problems in active smokers and people around their proximity. Over 4800 compounds in the particulate matter and vapour phases of the cigarette smoke are considered to play crucial roles in the pathophysiology of nearly all the smoking-related disorders in adults, such as cardiovascular diseases, immune dysfunctions, and cancer [1,2]. Many of these detrimental effects of smoking result from severe oxidative damages to critical biological entities. The aggravation of oxidative stress can even trigger apoptosis and necrosis in different cell types, and get involved in many diseases, such as atherosclerosis, Parkinson’s disease, Alzheimer’s disease, etc. [3,4]. Pregnancy is a physiological state in itself, which is associated with an increased metabolism and oxygen demand [5]. The intrauterine hypoxic condition itself induces the generation of free radicals and foetal oxidative stress [6]. Moreover, a maternal smoking habit acts as an additional stressor, increasing the foetal oxidative/nitrosative stress due to the toxic components of cigarette smoke (like lead, nicotine, cotinine, cyanide, cadmium, mercury, carbon monoxide and polycyclic aromatic hydrocarbons, etc.), unfiltered by the placenta [7,8]. Such harmful substances have a primary impact on the circulating blood cell population and the umbilical cord vein, carrying oxygen-rich blood [9,10,11]. However, it is important to mention that a protective effect of substances entering/forming the body during smoking is also assumed against the development of certain abnormalities during pregnancy. Several studies have shown that the risk of developing preeclampsia is lower for women who smoke. This observation can be related to the elevated CO level exposure [12,13].

The key functions of the circulating blood cells include the transport of oxygen and waste carbon dioxide, the regulation of homeostasis (thermoregulation, buffer capacity… etc.), and protection against infections, foreign cells, and proteins. Over a long period of time, red blood cells (RBCs), being the major population of the total blood, were taken as carriers for tissue oxygenation, responsible for the transmission of metabolic gases, nutrients and used as a sink for nitric oxide (NO). Our perception towards the general role of RBCs has undergone a paradigm shift in the last decade, and there are emerging evidences that they may also play significant roles in the functional regulation of the endothelial cells and even can mediate endothelial dysfunction. A new functional role of RBCs was stated, according to Kelm and colleagues, that the adult RBCs are not only passive regulators of NO levels from the endothelium, but erythrocytes themselves are also able to synthesize and export NO, thus helping to maintain vascular tone and blood flow [14,15]. Additionally, RBCs act as vital sensor entities in response to the wide range of vascular/endothelial dysfunctions/diseases. Moreover, their changed protein-profile might even augment and can synergize the prognosis of vascular dysfunction/comorbidities [16]. Any changes in the RBC phenotype along with their rheological characteristics could serve as a primary indicator for their altered functionality. In our previous work, we had described how maternal smoking induced significant alterations in the morphological parameters, elasticity, and plastic properties of foetal RBCs and in this same context, we have also highlighted the altered composition of their membrane-forming lipids [10,16]. Oxidative stress increases the radical-mediated peroxidation of ω-6 polyunsaturated fatty acids inducing the formation of one of the primary lipid’s peroxidized product 4-Hydroxy-trans-2-nonenal (4-HNE). 4-HNE is highly capable of binding to glutathione (GSH), resulting in a decreased antioxidant capacity [17], and additionally, it also plays a key role in the regulatory mechanisms responsible for GSH biosynthesis, insulin secretion, and at higher concentrations, 4-HNE is even involved in the pathogenesis of several inflammatory-based diseases [18].

RBCs’ membrane plays an important role in maintaining the structure, biological transport, and homeostasis of the cells mediated by unique proteins with specific functions. Band 3, an integral membrane protein that accounts for almost a quarter of the surface of the RBC membrane, takes parts in the anion transport across the membrane, regulation of the glycolytic pathway, stabilization of the membrane structure, and the control of the RBC lifespan [19]. At the physiological condition, several Band 3 molecules are bound to spectrin, and co-ordinates the well-orchestrated cytoskeletal network. However, an increase in the oxidative stress level results in Band 3 phosphorylation and further dissociation from the spectrin cytoskeleton [20].

In our previous studies, one of the main characteristic features behind the maternal smoking-induced morphological and functional alterations was the development of oxidative stress by the increased amount of free radical formation. In the case of a fungal infection, the host defence system is mobilized against the intruder and one of the key mechanisms provided by innate immune cells also imply the increased generation of free radicals [21]. Based on recent statistical data, nearly 2 million patients/year suffer an acute invasive fungal infection [22], and more than 50% of it can be attributed to *Candida* species, mostly to *Candida albicans* (*C. albicans*) but a rapid increase in the prevalence of non-albicans *Candida* species, i.e., *Candida parapsilosis* (*C. parapsilosis*) has also been observed in the past decades [22,23]. Invasive *Candida* infections pose a significant health threat today, particularly for individuals with compromised immune systems, i.e., heavy smokers, and neonates with a low birth weight. The aim of the study was to examine the effects of smoking on maternal and foetal RBCs. We sought to answer how long-term stress on RBCs affects their ability to respond to an acute insult. On the one hand, we planned to explore the effect of *Candida* infection, as a secondary stressor, on the integrity, morphological, and molecular properties of maternal and foetal RBCs with markedly different redox capacities. On the other hand, we also wanted to examine the effect of RBCs with different origins on the survival potential of the two *Candida* species. 

## 2. Results

### 2.1. Hemolytic Activities of C. parapsilosis and C. albicans on Whole Blood and Isolated Red Blood Samples

**Figure 1:**
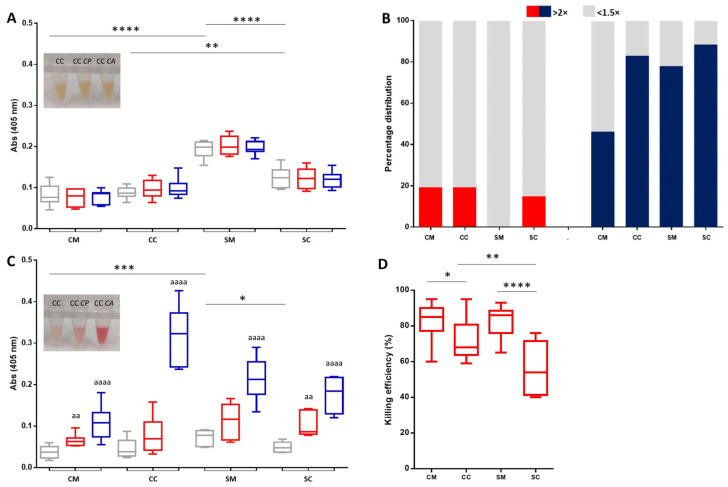
Graphical summary showing the hemolytic activity of *Candida* spp. on the maternal and foetal blood samples. Hemolytic activity of *C. parapsilosis* (red) and *C. albicans* (blue) was measured on both the foetal and maternal whole blood (**A**) and isolated red blood cell populations (**C**), with smoking and non-smoking backgrounds. Absorbance of *Candida*-free controls are indicated by the grey boxes, *n* = 16 for smoking and 25 for non-smoking groups. Minimized pictorial inserts on panel (**A**,**C**) indicate the colour of supernatants originating from the foetal blood samples with and without the *C. parapsilosis* (CC *CP*) and *C. albicans* (CC *CA*) infection. The hemolytic-factor production by *Candida* strains on whole blood populations is represented in the panel (**B**). The hemolytic activities of cell-free supernatants, originating from *C. parapsilosis* (red)- and *C. albicans* (blue)-infected total blood, were tested on the corresponding isolated red blood cell population. Each column indicated the percentage of the sample population with significant hemolytic activity (*n* = 25 for CM and CC, and *n* = 16 for SM and SC). The measurement of the fungicidal efficiency in whole blood (**D**) by CFU-determinations after 20 h of *C. parapsilosis* infection; *n* = 25 for non-smoking, and *n* = 16 for smoking groups. CM: control (non-smoking) mother, CC: control child, SM: heavy smoker mother, SC: child born to smoking mother, CFU: colony-forming unit. The results were accepted to be significant by statistical analyses using one-way ANOVA and the Newman–Keuls multiple comparison test or unpaired *t*-test at * *p* < 0.05, **^/aa^ *p* < 0.01, *** *p* < 0.001, and ****^/aaaa^ *p* < 0.0001. * indicate the significant differences among the various untreated groups (CM, CC, SM, and SC), and ^a^ between the untreated control and *Candida*-infected samples.

The hemolytic activity of *C. parapsilosis* and *C. albicans* was tested on the foetal and maternal blood samples originating from smoking and non-smoking populations. Whole blood and isolated RBCs with a different origin were infected with a 1/50 volume of fungal suspensions, resulting in an infectious dose of 1 × 10^7^/mL. Additionally, with this experimental set up, we can also gain information on the immunity status of non-smoking and smoking sample groups against the two different yeast populations. In the case of whole blood, a significant difference in the cell lyses was observed only between the uninfected sample groups after 20 h of incubation at 37 °C. The highest level of hemolysis was measured in the SM samples with ~3-fold differences to the CM population. However, neither the *C. parapsilosis* nor the *C. albicans* infections significantly affected the basal hemolytic level in any of the sample groups (Figure 1A).

*Candida* spp., to support their survival rate, may acquire iron from RBCs by processing a hemolytic factor that promotes cell lysis. Therefore, a production of the lysis factor can be assumed, despite the fact that we have not observed any significant increase in the hemolysis pattern upon the infection of the whole blood. To test this hypothesis, hemolytic activities of cell-free supernatants, originating from *C. parapsilosis-* and *C. albicans*-infected total blood, was followed on corresponding RBC isolates. In the case of the supernatants of *C. parapsilosis*-infected whole blood, 80–100% of each sample populations showed only a minimal increase in cell lysis (less than 1.5-fold), compared to their negative control. On the contrary, a significantly higher hemolytic activity (2–2.5-fold increase) was obtained in all the tested groups using a supernatant of the *C. albicans*-infected blood. The sample population originating from the non-smoking mother proved to be highly resistant (only 45% of the populations exhibited a 2–2.5-fold increase in cell lyses), while the other three groups (CC, SM, and SC) exhibited a significantly higher sensitivity, and 80~90% of the samples showed an increased level of hemolysis (Figure 1B).

We reached a similar conclusion by investigating directly the effect of fungal infection on the isolated RBCs. Without the protective effect of the immune system, *C. albicans* have induced cell lysis with a higher efficiency on the RBCs in comparison to *C. parapsilosis* (Figure 1C). The most intensive hemolytic effect was induced on the foetal population with a non-smoking origin (~6.5 fold), while there is no significant difference in the induction levels in the cases of the CM, SM, and SC sample groups (3–3.3-fold) (Figure 1C).

In connection to the hemolysis tests, the effect of the blood samples on the survival rate of the fungi was also investigated. In whole blood, the defence system against a yeast infection was activated, and we found that the maternal samples have the highest killing efficiency on the *C. parapsilosis*, regardless of the sample origin. In case of neonates, significant differences relative to each other and to maternal populations (CC/SC CC/CM, and SC/SM sample pairs) were detected, where neonates with a smoking origin showed the lowest killing efficiency (Figure 1D).

### 2.2. Candida Infection-Induced Morphological and Molecular Alterations in the Membrane Integrity of Red Blood Cell Populations

Although the whole blood infected with *Candida* strains did not show an increased hemolytic pattern, routine eosin staining of blood smears revealed severe morphological changes and/or aberrations in the red blood cells, suggesting that the cells may have suffered damage that could modify their integrity and function. Therefore, we looked for the two *Candida* strains that induced characteristic changes among the sample groups with different origins. Firstly, we investigated the appearance of special morphological variants on eosin stained smears, secondly, we searched for possible rearrangements of membrane proteins with crucial functions in homeostasis and cell shape by the immunocytochemistry technique using the Band 3-specific antibody, and thirdly, we checked the status of the membrane lipid layer by measuring the accumulation of 4-HNE, the molecular marker for the lipid peroxidation.

**Figure 2:**
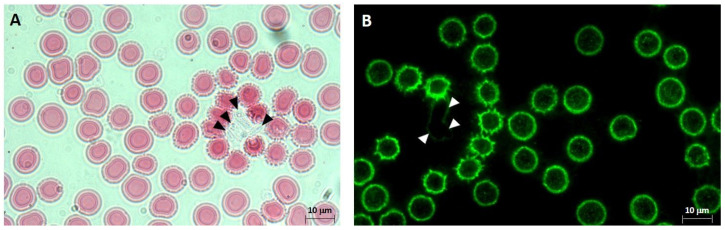
*C. parapsilosis*-induced morphological changes in the red blood cell. Representative micrographs of eosin-stained (**A**) and immuno-labelled (**B**) *C. parapsilosis*-infected maternal whole blood with a non-smoking origin. *C. parapsilosis* is indicated with triangles marks. Immuno-labelling was done with a monoclonal mouse anti-CD235a (glycophorin) antibody with a 1:100 dilution, followed by an Alexa 488 (green) anti-mouse secondary antibody with a 1:1000 dilution. Slides were examined using a Nikon Eclipse 80i epifluorescence microscope with an 100× immersion objective.

In our previous publications, we presented evidence that maternal smoking, heavy metal treatment, or by mimicking *C. parapsilosis* infection significantly increased the appearance of morphological variants, especially the echinocyte type, in the foetal blood samples. Here, we mainly looked for *Candida* spp.-induced characteristic phenotypical changes, i.e., clustered populations with shrinkage and membrane blebbing, in both the maternal and foetal total blood population with non-smoking and smoking origins. We checked a fairly high number of independent sample groups with and without *Candida* infections (19 of CM/CC, and 14 of SM/SC populations) and found no significant amounts of such RBC cluster formations in the uninfected samples, including samples with a smoking origin. *C. parapsilosis* and *C. albicans* infections induced cluster formations in all the four sample groups, regardless of the type of *Candida* spp. The number of clusters was ~2–4/μL of blood in the case of CM, CC, and SM samples, while it was significantly higher in children born to smoking mothers; ~4–6/μL of the sample. The most interesting thing about this type of cluster formation was the presence of fungus in the centre or at the vicinity of the clusters (Figure 2).

**Figure 3:**
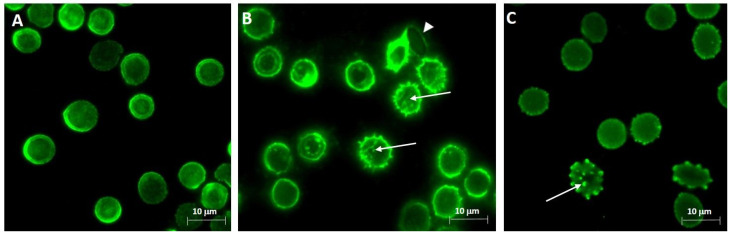
Visualization of Band 3 distribution pattern in the RBCs. Representative epifluorescent micrographs of Band 3 distribution in the control (**A**) and *C. parasilosis*-infected (**B**,**C**) maternal RBCs with a non-smoking origin. White arrows indicate the clustered Band 3 proteins on RBC membranes. *C. parapsilosis* is represented by a white arrow mark on the panel (**B**). Immuno-labelling was performed using the monoclonal mouse anti-Band 3 antibody with a 1:100 dilution, followed by an Alexa 488 (green) anti-mouse secondary antibody with a 1:1000 dilution. Micrographs were captured using a Nikon Eclipse 80i epifluorescence microscope with an 100× immersion objective.

In parallel, with the cluster formation of deformed RBCs, we also detected an alteration in the Band 3 pattern. Here, we followed Band 3 localisation by immunocytochemistry using specific antibody for the protein and looked for an alteration in the distribution/cluster formation of Band 3. It seems that Band 3 loses its connection to the cytoskeletal network and lines up in clusters in some morphological altered cells (Figure 3A–C). The uninfected samples, as derived from the non-smoking mothers and their respective neonates, showed altered Band 3 patterns in ~2.5–4% of the cells, while in samples with a smoking origin, the clustering of Band 3 was detected in ~8% of the cells. After fungal infection, these values were increased 3.5–4 and 1.5–2-fold in non-smoking and smoking sample groups, respectively, with the exception of the *C. albicans*-infected control foetal populations, where we detected a ~5–5.5-fold increase in the affected cell number.

**Figure 4:**
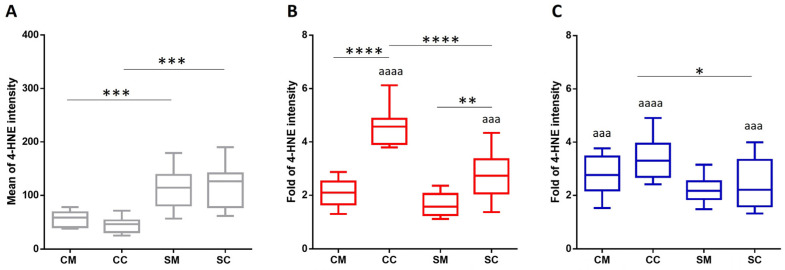
Quantification of the 4-HNE level in foetal and maternal RBC populations. RBCs were isolated from the total blood without (**A**) and with a *C. parapsilosis* (**B**) or *C. albicans* (**C**) infection for 20 h. Significant changes in the fluorescent intensity were determined by FACS analysis. Immuno-labelling was done using a monoclonal mouse anti-4-HNE antibody with a 1:100 dilution, followed by an Alexa 488 anti-mouse secondary antibody labelling with a 1:1000 dilution. The 4-HNE levels were compared to the corresponding uninfected samples that were kept under the same conditions. The sample numbers were *n* = 9 for each of the examined groups. CM: control (non-smoking) mother, CC: control child, SM: heavy smoker mother, SC: child born to smoking mother. The results were accepted to be significant by statistical analyses using one-way ANOVA and the Newman–Keuls multiple comparison test or unpaired *t*-test at * *p* < 0.05, ** *p* < 0.01, ***^/aaa^ *p* < 0.001, and ****^/aaaa^ *p* < 0.0001. * indicate the significant differences among the increases of sample groups, and ^a^ between the untreated control and *Candida*-infected samples.

Membrane damage was further monitored by measuring the extent of lipid peroxidation. We applied immunolabelling to follow one of the major lipid peroxidation end products, 4-HNE, the α,β-unsaturated hydroxyalkenal. A significant difference was measured in the level of 4-HNE between the non-smoking and smoking sample populations. Compared to the corresponding non-smoking groups, the SM and SC samples showed ~2 and ~2.5 times higher values, respectively. Following *Candida* infection, a significant increase was detected in all the investigated groups except the smoking mother population; 2–2.5-fold in CM, 5–3.5 in CC, 1.5–2 in SM and ~2.5–2 in the case of SC, induced by *C. parapsilosis* and *C. albicans,* respectively (Figure 4).

### 2.3. The Activation of Caspase Cascade and Complement System by Fungal Infection

**Figure 5:**
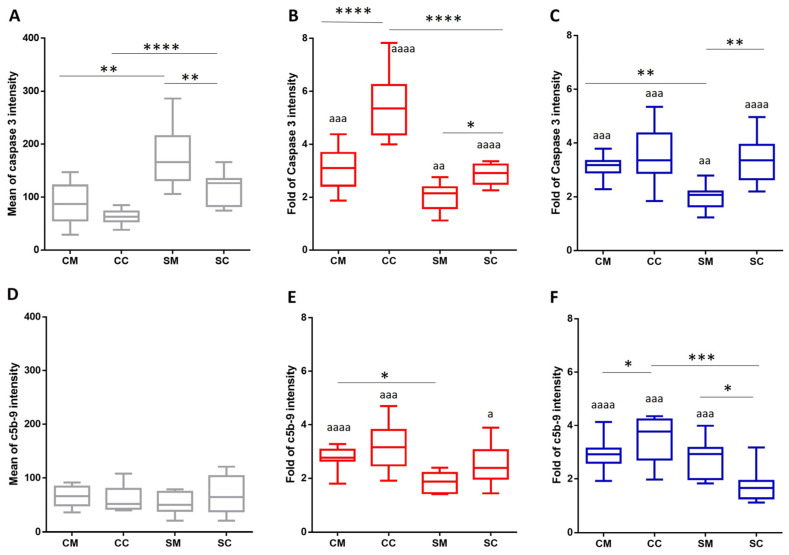
Graphs quantifying the active Caspase 3 (**A**–**C**) and c5b-9 levels (**D**–**F**) in and on foetal and maternal RBCs. RBCs were isolated from whole blood without (**A**,**D**) and with *C. parapsilosis* (**B**,**E**) and *C. albicans* (**C**,**F**) infections. Changes in the fluorescent intensity were determined by FACS analysis. Immuno-labelling was performed using a rabbit anti-Caspase 3 and mouse c5b-9 antibody with a 1:100 dilution, and followed by Alexa 488 or 647 secondary antibodies labelling with a 1:1000 dilution. The mean fluorescence intensity levels were compared to the same uninfected sample kept under similar treatment conditions. The sample numbers were *n* = 9 for each of the examined group. CM: control (non-smoking) mother, CC: control child, SM: heavy smoker mother, SC: child born to smoking mother. The results were accepted to be significant by statistical analyses using one-way ANOVA and the Newman–Keuls multiple comparison test or unpaired *t*-test at *^/a^ *p* < 0.05, **^/aa^ *p* < 0.01, ***^/aaa^ *p* < 0.001, and ****^/aaaa^ *p* < 0.0001. * indicate the significant differences among the sample groups, and ^a^ between the untreated control and *Candida*-infected samples.

In the case of RBCs from an arsenic-treated rat, Shen et al., has identified Caspase 3 as primary mediator for the 4-HNE-induced cellular damage [24]. Here, we followed the 4-HNE-protein adducts-initiated death signal cascade by measuring the active Caspase 3 in RBC populations isolated from the total blood with and without fungal infection. For quantification, the immuno-labelled active Caspase 3 was measured by the fluorescence-activated cell sorting (FACS) method. The smoking sample groups had nearly twice the active Caspase-3 levels as the maternal and foetal control populations (Figure 5A). All four sample groups showed a significant, minimum 2-fold increase, irrespective of the yeast species. The highest elevation was induced by *C. parapsilosis* (over 5-fold) in the foetal sample with a non-smoking origin (Figure 5B,C).

In parallel to the apoptotic marker Caspase 3, we also followed the presence of the terminal c5b-9 complement complex in all of the four sample groups. The c5b-9 complex, formed during the *Candida* infection-initiated activation of the host complement system, gets deposited on the cell surface and might also affect RBC survival. A significant increase was measured in the samples with a non-smoking origin (CM and CC), irrespective of the *Candida* spp.. The highest level of c5b-9 deposition was detected on the foetal samples (3–4-fold). The deposition of c5b-9 in the maternal and foetal samples with a smoking origin lagged behind the non-smoking values (Figure 5E,F).

## 3. Discussion

The major aim of this study was to follow the consequences of fungal exposure, as a secondary stressor, on the already compromised RBC populations. The effect of the *Candida* infection was investigated on four blood sample groups (mothers and their neonates originating from non-smoking versus smoking populations) with marked differences in the level of reactive oxygen species, the capacity of the antioxidant and immune systems, and the morphological and rheological parameters of the RBCs.

Our previous studies have highlighted that one of the main characteristic features behind the maternal smoking-induced morphological and functional alterations in the RBCs was due to the development of oxidative stress by the excessive free radical load. However, the accumulated toxic substances due to smoking, specifically the strong oxidants such as peroxynitrite, might also have a prompt action on the invading fungal pathogens. Based on such assumptions, the main findings of this present study are as follows: (a) The difference in the baseline hemolysis of whole blood originating from control and smoker populations is due to smoking, and acute *Candida* infection does not increase it further. Fungal infections induced similar rates of hemolysis on all the isolated RBC sample groups, except the control foetal cells. (b) The appearances of distinct morphological alterations such as cluster formations with shrunken, membrane blebbing cells exclusively gets related to the fungal infection. (c) In contrast, the formation of Band 3 clusters and 4-HNE adducts occurred in the sample origin (maternal versus foetal) and the *Candida* spp. specific manner. (d) There lies no major difference in the induction level of Caspase 3 between smoking and non-smoking sample groups with the only exception of *C. parapsilosis* infected foetal sample group.

Both in the whole blood and the isolated RBC samples with smoking origins, a higher baseline for hemolysis compared to the samples from non-smoker individuals was shown. Among all the possibilities, firstly it should be mentioned that due to the accumulation of toxic materials, smoking induces nano hemolysis as nano ruptures occur on the cell surface [9]. Similarly, cotinine, unfiltered by the placenta, results in an elevated level of lipid peroxidation [25] and further increases the hemolytic values. Furthermore, RBCs’ membrane has a principal role in maintenance of the homeostatic condition of the cells. Smoking induces a higher distribution of the RBC phenotypic variants, where, among others, the stomatocytosis and spherocytosis, due to the increased cell volume and decreased elasticity of the membrane, might increase the fragility of the cells [26].

It seems that the acute ex vivo *Candida* infection did not increase the rate of cell lyses in whole blood populations, not even in sample groups with the smoking background. Similarly, the infections have not shown a more drastic effect on the isolated maternal RBCs derived from the smokers with respect to the control samples. In addition to the health-damaging effects of substances accumulated in the smoking sample groups, they reduce the risk of preeclampsia [12,13]. Similarly, a wide range of toxic compounds have characteristic protective or destructive effects on fungal survival. Based on the in vitro studies, nicotine can significantly increase the *C. albicans* and *C. parapsilosis* adhesion and growth [27]. On the other hand, fungal growth can be effectively inhibited by human serum. Here, the underlying fact is that a *Candida* growth gets directly dependent on the iron saturation of the serum [28]. In the human serum, obtained from the smoking background, the level of the iron-binding protein transferrin gets increased; hence, as a result, there is no excess extracellular iron in the system which would support *Candida* survival [29].

In another perspective, there lies a significant increase, among the smokers, in the white blood cell count, including the neutrophils and macrophages, and furthermore even the T and B lymphocytes are also affected [30]. Hence, in addition to the rise of the innate immune response, the adaptive immune system is also activated well before the fungal infection [31,32,33]. It could be important for the yeast survival, especially in the case of smoking-induced iron-deficiency anaemia [34]. Furthermore, it has also been observed that cigarette smoke pre-treatment of *C*. *albicans* increased the resistance against osmotic and heat stresses and significantly increased the chitin content of yeast, particularly under hyphae culture conditions [35]. Besides, the impact of smoking-derived toxic compounds on the regulatory system induces a hyper activation of the complement cascade, resulting in the exhaustion of the complement system by causing a fast depletion of the C3 and C4 complement factors [36].

The physiological aging of RBCs is accompanied by morphological changes [37]. The molecular backgrounds behind these alterations, among others, are the oxidative modification and cross-links formation between the Band 3 transmembrane proteins. This process prepares the aging cells for opsonisation and their removal from the system [38,39]. The increased appearance of certain types of morphological variants along with the modification and rearrangement of the Band 3 proteins can also be linked to external stressors as induced by the free radical formation [16,40]. This phenomenon can be directly connected to smoking; for both the maternal and the foetal RBCs originating from the smoking populations, the number of cells with altered Band 3 patterns was at least 2.5 times that of the control samples. *Candida* infection was also able to increase the altered distribution of Band 3, both in smoking and non-smoking groups. The degree of change caused by the *Candida* infection differed significantly between the two groups. Control samples were more affected, which suggests that the smoker-derived samples may have partially adapted to the “toxic” environment as a result of a previous exposure to the free radical attack. Further, it also indicates that the changed Band 3 pattern is not a unique phenotypical characteristic for the fungal infection.

The direct connection between the eryptosis-based cell death and smoking still remains unclear [41,42]. Eryptosis is a special mechanism to remove the injured RBCs from the circulation prior hemolysis. Eryptosis can be stimulated by a variety of stressors: oxidative stress, hyperosmotic shock, hyperthermia, energy depletion, and a wide range of endogenous substances [43]. In the majority of the smoking samples as examined in our previous and current work, the characteristic phenotype of eryptosis/apoptosis, the shrunken, membrane blebbing and clumping of RBCs, was not typical. After the acute fungal infection, however, these phenotypic variants appeared in all four sample groups. Additionally, the *Candida* infection increased the 4-HNE adduct formation and Caspase 3 activation with a minimum of a 2-fold, regardless of the sample origin. Further, activation of the Caspase 3 enzyme mimicked the pattern of 4-HNE formation. It is more likely that the Caspase 3 is a mediator of 4-HNE-induced cell death/eryptosis in all four sample groups. A similar result was reached by Allegra and co-workers when they verified, in an ex vivo experiment, that a 4-HNE treatment of RBCs resulted in the Caspase 3 activation-mediated eryptosis [18]. In the course of our present and earlier work, we have experienced that smoking itself also induces the formation of 4-HNE, the activation of Caspase-3, and the increased appearance of phosphatidylserine on the outer surface of the RBC membrane [44,45]. However, the substantial cell volume reduction and membrane blebbing were nevertheless not typical in the SM and SC samples. The emergence of this phenotype can clearly be attributed to the fungal infection-induced high-active Caspase 3 level and the c5b-9 complex deposition on the cell surface.

The major strength of this study is the potential of the sample populations. On the one hand, in addition to the comparative analysis of the two main groups, non-smoker and smoker populations, it also provides an opportunity to follow the consequences of maternal smoking in the mother–fetus (neonate) relationship. On the other hand, it provides an opportunity to investigate how a long-term exposure influences the ability to respond to an acute stress situation.

## 4. Materials and Methods

### 4.1. Human Sample

According to Declaration of Helsinki, the umbilical cord blood and adult whole blood derived from the vein of heavy smoker (at least 10 cigarettes per day) and non-smoker pregnant mothers were collected from the Department of Obstetrics and Gynaecology, University of Szeged, Hungary. The study protocol (114/2020) was approved by the Ethics Committee of the Department of Obstetrics and Gynaecology, University of Szeged, Hungary. Considering the informed consent, all the clinical samples were collected based on the following inclusion criteria: (i) average age of the mothers must be around 30 years (ii) they must not possess or suffer from any type of congenital anomalies, long-term or pregnancy-induced metabolic disorders (iii) healthy neonate born no earlier than the 37th week of gestation (iv) an uncomplicated pregnancy status.

### 4.2. Candida Infection

After the collection of the fresh blood samples within 2 h, they were incubated for almost 20 h at a temperature of 37 °C in a CO_2_ thermostat (5%) as the control samples (i.e., without *Candida* infection) and the rest of the samples were infected either with *Candida parapsilosis* (ATCC 22019) or *Candida albicans* (SC 5314). For the maintenance of the *Candida* species, the YPD plates (1% yeast extract, 2% bactopeptone, and 2% glucose and 2.5% agar) were used which were initially kept at 4 °C. Before the start of the experiment, the *Candida* species were grown in a liquid YPD medium (1% yeast extract, 2% bactopeptone, and 2% glucose) overnight at a 37 °C temperature in a shaker incubator. After the incubation period, the cells were washed twice with PBS (pH 7.4), and counted in a Bürker chamber with the final concentration of 10^7^ fungi/mL of the total blood. To check the killing efficiency, 50 µL of the 1000× diluted suspension was again plated onto the YPD agar plates in triplicates and incubated at 30 °C for 2 days. The killing efficiency was calculated in % by the stated formula: [(number of living *Candida* cells in control wells − number of living *Candida* cells in co-cultures)/number of living *Candida* cells in control wells] × 100 [46]. Further, the infected blood samples were divided into 3 parts for the subsequent microscopic analysis, hemolysis test, and the FACS analysis.

### 4.3. Hemolytic Activity

The hemolytic activity recorded the effects of direct *Candida* infections in the whole blood as well as in the purified RBC population. Investigations were also done to check the effects of the cell-free supernatants derived from the whole blood after the *Candida* infection on the purified RBCs.

RBC samples were prepared by centrifugation at 200 rpm for 5 min. The lowest 2/3rd portion of the centrifuge was collected and washed with 2 volumes of the isotonic saline solution at pH 7.0 and finally the RPMI 1640 medium (Biowest, Nuaillé, France) twice the volume was added to the isolated and purified RBC for dilution at a ratio of 1:2 (*v*/*v*). The diluted RBC and the whole blood samples were then further infected and incubated for 20 h with *Candida* spp. (final concentration 10^7^/mL) under the specific conditions at 37 °C and 5% CO_2_. Examining the direct effects of *Candida* spp. infection, it is further subjected to a centrifuge at 1000 rpm, for 2 min at room temperature. The supernatant was collected and by the UV spectrophotometric measurements, the absorbance was measured at 405 nm (Genesys 10S UV-VIS, ThermoFisher Scientific, Madison, WI, USA) [47].

To determine the hemolytic activity of the cell-free supernatant, the purified RBC phase was diluted with the RPMI 1640 medium with the final concentration of 10^7^ cells/50 µL. To perform this, an addition of the whole blood supernatant with a direct *Candida* infection at a 1:1 (*v*/*v*) ratio was completed and incubated for 20 h under the specified conditions at 37 °C and 5% CO_2_ and further centrifuged to 1000 rpm for 2 min at room temperature. After centrifugation, the supernatant was collected and the absorbance was measured at 405 nm. The hemolytic activity in terms of percentage (%) was measured as per the formula: hemolysis (%) = 100 − [(Ap − As)/(Ap − An) × 100)], where Ap, As, and An are the absorbance of the positive, test, and the negative control, respectively [48]. Here, the negative controls were the supernatants of the *Candida*-infected whole blood without the RBCs, and the positive controls were the hemolysed RBCs with a 1:10 *v*/*v* in the H_2_O. Every time, the hemolytic activity (%) of the infected supernatant was compared to the effect of the uninfected supernatant sample under the same optimised conditions.

### 4.4. Fluorescence-Activated Cell Sorting (FACS) Analysis

At first, a fixation of the RBCs with 4% (*w*/*v*) paraformaldehyde at 4 °C for 60 min was performed. This was followed by the consecutive washing step in a 0.05 M phosphate buffer (PB) at pH 7.4. The RBCs were permeabilized with 0.1% of Triton-100X for the duration of 20 min and further incubated for 1 h in PB containing 1% bovine serum albumin and 10% normal goat serum to block the non-specific antibody binding. After the blocking step, incubation was performed with the primary antibodies by single or double staining using the rabbit anti-Cleaved Caspase 3 antibody (ab2302, Abcam, Cambridge, UK); mouse anti 4-hydroxy-2-nonenal antibody (4-HNE) (ab48506, Abcam, Cambridge, UK); and mouse anti-C5b-9 antibody (aE11), (sc-58935, Santa Cruz Biotechnology Inc., Dallas, TX, USA), at 4 °C overnight. Subsequently, the secondary antibodies with the goat anti-mouse Alexa^®^647 (ab150115, Abcam, Cambridge, UK) and/or with the goat anti-rabbit Alexa^®^488-conjugated (ab150077, Abcam, Cambridge, UK) for 1 h in the dark at room temperature were added and the RBCs were further processed for the quantitative analysis (FACS, BD FACS Calibur™, BD Biosciences, Franklin Lakes, NJ, USA). The FACS data were analysed using the FlowJo_V10 software tool.

### 4.5. Microscopic Analysis

Immunocytochemistry was performed on the fixed blood smears (150 °C, 20 min), where permeabilization was done by 0.1% Triton-100X for 30 min and then incubated for 1 h in PB containing 1% bovine serum albumin and 10% normal goat serum to block the non-specific antibody binding. Incubations with the primary antibodies using mouse anti-CD235a antibody (JC159), (MA5-12484, Thermo Fisher Scientific, Madison, WI, USA) and mouse anti-Band 3 antibody (A-6), (sc-133190, Santa Cruz Biotechnology Inc., Dallas, TX, USA), by single staining were performed at 4 °C overnight, and were followed by the secondary antibody goat anti-mouse Alexa^®^488 (ab150113, Abcam, Cambridge, UK) for 1 h at room temperature. In parallel, eosin-stained (HT110316; Sigma Aldrich, Saint Louis, MO, USA) blood smears of the infected samples were also examined to detect the morphological changes in the RBCs. Finally, the prepared slides were mounted using the Immunohistomount (Sigma Aldich, Saint Louis, MO, USA) and were visualised under an epifluorescence microscope (Nikon Eclipse 80i, 100× immersion objective; Nikon Zeiss Microscopy GmbH, Jena, Germany); pictures were taken with a QImaging RETIGA 4000R camera, using Capture Pro 6.0 software (QImaging, Surrey, BC, Canada).

### 4.6. Statistical Analyses

All statistical analyses were performed using the GraphPad Statistical Software version 4.0. The minimum to maximum values were presented as the median. In comparison between the two groups, we used only the unpaired *t*-test, or else all other statistical analyses were done by the one-way analysis of variance (ANOVA) using the Newman–Keuls multiple comparison test. The significant differences were accepted at * *p* < 0.05, ** *p* < 0.01, *** *p* < 0.001, and **** *p* < 0.0001.

## 5. Conclusions

A major part of the vascular interactome deals with RBC homeostasis and its crucial functionalities. This study highlights the phenotypical and molecular features of the RBC population under intensive stress conditions, such as sustained smoking during pregnancy aggravated by the acute *Candida* infection. We intended to point out, that in this case, the fungal infection synergized the smoking-induced adverse symptoms, like appearance of morphological variants, the formation of the lipid peroxidation marker 4-hydroxyl-nonenal, and the activation of Caspase 3. The *Candida*-infected control RBCs mimicked the characteristic features of smoking sample populations, indicating a general stress response, probably due to the altered redox state of the cells. Additionally, we were also able to identify an atypical phenotype-clustered population with shrinkage and membrane blebbing, in both the non-smoking and smoking populations, which can be exclusively linked to the fungal infection. In the immediate vicinity of these clusters, the presence of the fungus was detected; therefore, it can be highly assumed that secreted fungal proteins might play a role in the development of this phenotype. This hypothesis requires further studies in the future.

## Data Availability

Data supporting the reported results are available upon request.

## References

[1] Feltes B.C., de Faria Poloni J., Notari D.L., Bonatto D. (2013). Toxicological effects of the different substances in tobacco smoke on human embryonic development by a systems chemo-biology approach. PLoS ONE.

[2] McGrath-Morrow S.A., Gorzkowski J., Groner J.A., Rule A.M., Wilson K., Tanski S.E., Collaco J.M., Klein J.D. (2020). The Effects of Nicotine on Development. Pediatrics.

[3] Pizzino G., Irrera N., Cucinotta M., Pallio G., Mannino F., Arcoraci V., Squadrito F., Altavilla D., Bitto A. (2017). Oxidative Stress: Harms and Benefits for Human Health. Oxid. Med. Cell. Longev..

[4] Jomova K., Raptova R., Alomar S.Y., Alwasel S.H., Nepovimova E., Kuca K., Valko M. (2023). Reactive oxygen species, toxicity, oxidative stress, and antioxidants: Chronic diseases and aging. Arch. Toxicol..

[5] Bizoń A., Milnerowicz-Nabzdyk E., Zalewska M., Zimmer M., Milnerowicz H. (2011). Changes in pro/antioxidant balance in smoking and non-smoking pregnant women with intrauterine growth restriction. Reprod. Toxicol..

[6] Buonocore G., Perrone S. (2006). Biomarkers of oxidative stress in the fetus and newborn. Hematol. Rep..

[7] Morgan J.C., Byron M.J., Baig S.A., Stepanov I., Brewer N.T. (2017). How people think about the chemicals in cigarette smoke: A systematic review. J. Behav. Med..

[8] Cha S.R., Jang J., Park S.M., Ryu S.M., Cho S.J., Yang S.R. (2023). Cigarette Smoke-Induced Respiratory Response: Insights into Cellular Processes and Biomarkers. Antioxidants.

[9] Masilamani V., AlZahrani K., Devanesan S., AlQahtani H., AlSalhi M.S. (2016). Smoking Induced Hemolysis: Spectral and microscopic investigations. Sci. Rep..

[10] Balogh G., Chakraborty P., Dugmonits K.N., Péter M., Végh A.G., Vígh L., Hermesz E. (2020). Sustained maternal smoking-associated changes in the physico-chemical properties of fetal RBC membranes might serve as early markers for vascular comorbidities. Biochim. Biophys. Acta Mol. Cell Biol. Lipids.

[11] Zahorán S., Szántó P.R., Bódi N., Bagyánszki M., Maléth J., Hegyi P., Sári T., Hermesz E. (2021). Sustained Maternal Smoking Triggers Endothelial-Mediated Oxidative Stress in the Umbilical Cord Vessels, Resulting in Vascular Dysfunction. Antioxidants.

[12] Bainbridge S.A., Belkacemi L., Dickinson M., Graham C.H., Smith G.N. (2006). Carbon monoxide inhibits hypoxia/reoxygenation-induced apoptosis and secondary necrosis in syncytiotrophoblast. Am. J. Pathol..

[13] Wikström A.K., Stephansson O., Cnattingius S. (2010). Tobacco use during pregnancy and preeclampsia risk: Effects of cigarette smoking and snuff. Hypertension.

[14] Cortese-Krott M.M., Kelm M. (2014). Endothelial nitric oxide synthase in red blood cells: Key to a new erythrocrine function?. Redox. Biol..

[15] Pretini V., Koenen M.H., Kaestner L., Fens M.H.A.M., Schiffelers R.M., Bartels M., Van Wijk R. (2019). Red blood cells: Chasing interactions. Front. Physiol..

[16] Dugmonits K.N., Chakraborty P., Hollandi R., Zahorán S., Pankotai-Bodó G., Horváth P., Orvos H., Hermesz E. (2019). Maternal Smoking Highly Affects the Function, Membrane Integrity, and Rheological Properties in Fetal Red Blood Cells. Oxid. Med. Cell Longev..

[17] Caliri A.W., Tommasi S., Besaratinia A. (2021). Relationships among smoking, oxidative stress, inflammation, macromolecular damage, and cancer. Mutat. Res. Rev. Mutat. Res..

[18] Allegra M., Restivo I., Fucarino A., Pitruzzella A., Vasto S., Livrea M.A., Tesoriere L., Attanzio A. (2020). Proeryptotic Activity of 4-Hydroxynonenal: A New Potential Physiopathological Role for Lipid Peroxidation Products. Biomolecules.

[19] Jennings M.L. (2021). Cell physiology and molecular mechanism of anion transport by erythrocyte band 3/AE1. Am. J. Physiol. Cell Physiol..

[20] Shimo H., Arjunan S.N., Machiyama H., Nishino T., Suematsu M., Fujita H., Tomita M., Takahashi K. (2015). Particle Simulation of Oxidation Induced Band 3 Clustering in Human Erythrocytes. PLoS Comput. Biol..

[21] Drummond R.A., Gaffen S.L., Hise A.G., Brown G.D. (2014). Innate Defense against Fungal Pathogens. Cold Spring Harb. Perspect. Med..

[22] Fang W., Wu J., Cheng M., Zhu X., Du M., Chen C., Liao W., Zhi K., Pan W. (2023). Diagnosis of invasive fungal infections: Challenges and recent developments. J. Biomed. Sci..

[23] Trofa D., Gácser A., Nosanchuk J.D. (2008). *Candida parapsilosis*, an emerging fungal pathogen. Clin. Microbiol. Rev..

[24] Wang X., Mu X., Zhang J., Huang Q., Alamdar A., Tian M., Liu L., Shen H. (2015). Serum metabolomics reveals that arsenic exposure disrupted lipid and amino acid metabolism in rats: A step forward in understanding chronic arsenic toxicity. Metallomics.

[25] Asgary S., Naderi G., Ghannady A. (2005). Effects of cigarette smoke, nicotine and cotinine on red blood cell hemolysis and their -SH capacity. Exp. Clin. Cardiol..

[26] Won D.I., Suh J.S. (2009). Flow cytometric detection of erythrocyte osmotic fragility. Cytom. Part B.

[27] Gunasegar S., Himratul-Aznita W.H. (2019). Nicotine enhances the thickness of biofilm and adherence of *Candida albicans* ATCC 14053 and *Candida parapsilosis* ATCC 22019. FEMS Yeast Res..

[28] Ding X., Liu Z., Su J., Yan D. (2014). Human serum inhibits adhesion and biofilm formation in *Candida albicans*. BMC Microbiol..

[29] Caroline L., Taschdjian C.L., Kozinn P.J., Schade A.L. (1964). Reversal of serum fungistasis by addition of iron. J. Investig. Dermatol..

[30] Sherke B.A., Vadapalli K., Bhargava D.V., Sherke A.R., Gopireddy M.M.R. (2016). Effect of number of cigarettes smoked per day on red blood cell, lecocyte and platelet count in adult Indian male smokers—A case control study. Int. J. Med. Res. Health Sci..

[31] Qiu F., Liang C.L., Liu H., Zeng Y.Q., Hou S., Huang S., Lai X., Dai Z. (2017). Impacts of cigarette smoking on immune responsiveness: Up and down or upside down?. Oncotarget.

[32] Pedersen K.M., Çolak Y., Ellervik C., Hasselbalch H.C., Bojesen S.E., Nordestgaard B.G. (2019). Smoking and Increased White and Red Blood Cells. Arterioscler. Thromb. Vasc. Biol..

[33] Saint-André V., Charbit B., Biton A., Rouilly V., Possémé C., Bertrand A., Rotival M., Bergstedt J., Patin E., Albert M.L. (2024). Milieu Intérieur Consortium. Smoking changes adaptive immunity with persistent effects. Nature.

[34] Leifert J.A. (2008). Anaemia and cigarette smoking. Int. J. Lab. Hematol..

[35] Alanazi H., Semlali A., Perraud L., Chmielewski W., Zakrzewski A., Rouabhia M. (2014). Cigarette smoke-exposed *Candida albicans* increased chitin production and modulated human fibroblast cell responses. Biomed. Res. Int..

[36] Kokelj S., Östling J., Georgi B., Fromell K., Ekdahl K.N., Olsson H.K., Olin A.C. (2021). Smoking induces sex-specific changes in the small airway proteome. Respir. Res..

[37] Berezina T.L., Zaets S.B., Kozhura V.L., Novoderzhkina I.S., Kirsanova A.K., Deitch E.A., Machiedo G.W. (2001). Morphologic changes of red blood cells during hemorrhagic shock replicate changes of aging. Shock.

[38] Arese P., Turrini F., Schwarzer E. (2005). Band 3/complement-mediated recognition and removal of normally senescent and pathological human erythrocytes. Cell Physiol. Biochem..

[39] Gottlieb Y., Topaz O., Cohen L.A., Yakov L.D., Haber T., Morgenstern A., Weiss A., Chait Berman K., Fibach E., Meyron-Holtz E.G. (2012). Physiologically aged red blood cells undergo erythrophagocytosis in vivo but not in vitro. Haematologica.

[40] Pantaleo A., Ferru E., Pau M.C., Khadjavi A., Mandili G., Mattè A., Spano A., De Franceschi L., Pippia P., Turrini F. (2016). Band 3 Erythrocyte Membrane Protein Acts as Redox Stress Sensor Leading to Its Phosphorylation by p^72^ Syk. Oxid. Med. Cell. Longev..

[41] Attanzio A., Frazzitta A., Vasto S., Tesoriere L., Pintaudi A.M., Livrea M.A., Cilla A., Allegra M. (2019). Increased eryptosis in smokers is associated with the antioxidant status and C-reactive protein levels. Toxicology.

[42] Schmitt M., Ewendt F., Kluttig A., Mikolajczyk R., Kraus F.B., Wätjen W., Bürkner P.C., Stangl G.I., Föller M. (2024). Smoking is associated with increased eryptosis, suicidal erythrocyte death, in a large population-based cohort. Sci. Rep..

[43] Lang F., Lang E., Föller M. (2012). Physiology and pathophysiology of eryptosis. Transfus. Med. Hemother..

[44] Chakraborty P., Dugmonits K.N., Végh A.G., Hollandi R., Horváth P., Maléth J., Hegyi P., Németh G., Hermesz E. (2019). Failure in the compensatory mechanism in red blood cells due to sustained smoking during pregnancy. Chem. Biol. Interact..

[45] Zahorán S., Márton Á., Dugmonits K., Chakraborty P., Khamit A., Hegyi P., Orvos H., Hermesz E. (2022). Molecular Background of Toxic-Substances-Induced Morphological Alterations in the Umbilical Cord Vessels and Fetal Red Blood Cells. Int. J. Mol. Sci..

[46] Tóth A., Németh T., Csonka K., Horváth P., Vágvölgyi C., Vizler C., Nosanchuk J.D., Gácser A. (2014). Secreted *Candida parapsilosis* lipase modulates the immune response of primary human macrophages. Virulence.

[47] Sæbø I.P., Bjørås M., Franzyk H., Helgesen E., Booth J.A. (2023). Optimization of the Hemolysis Assay for the Assessment of Cytotoxicity. Int. J. Mol. Sci..

[48] Furlaneto M.C., Favero D., França E.J., Furlaneto-Maia L. (2015). Effects of human blood red cells on the haemolytic capability of clinical isolates of *Candida tropicalis*. J. Biomed. Sci..

